# CD39 Regulation and Functions in T Cells

**DOI:** 10.3390/ijms22158068

**Published:** 2021-07-28

**Authors:** Eleonora Timperi, Vincenzo Barnaba

**Affiliations:** 1 Department of Immunology, PSL Research University, INSERM U932, Institut Curie, 26, rue d’Ulm, 75005 Paris, France; 2 Department of Internal Clinical Sciences, Anaesthesiology and Cardiovascular Sciences, Sapienza Università di Roma, 00161 Rome, Italy; 3 Laboratory Affiliated to Istituto Pasteur Italia—Fondazione Cenci Bolognetti, 00161 Rome, Italy

**Keywords:** adenosine, CD39, CD73, regulatory T cells, conventional CD4^+^ T cells, CD8^+^ T cells, single nucleotide polymorphism, targeting therapy

## Abstract

CD39 is an enzyme which is responsible, together with CD73, for a cascade converting adenosine triphosphate into adenosine diphosphate and cyclic adenosine monophosphate, ultimately leading to the release of an immunosuppressive form of adenosine in the tumor microenvironment. Here, we first review the environmental and genetic factors shaping CD39 expression. Second, we report CD39 functions in the T cell compartment, highlighting its role in regulatory T cells, conventional CD4^+^ T cells and CD8^+^ T cells. Finally, we compile a list of studies, from preclinical models to clinical trials, which have made essential contributions to the discovery of novel combinatorial approaches in the treatment of cancer.

## 1. Adenosine Pathway and CD39/CD73 Expression in the Tumor Microenvironment

CD39 is an ectoenzyme (ecto-nucleotide triphosphate diphosphohydrolase 1, encoded by *ENTPD1* gene) belonging to the family of ectonucleotidases which comprises ecto-5′-nucleotidase (NT5E)/CD73, CD38/NADase, NAD glycohydrolases, nucleoside diphosphate kinase, ecto-nucleotide pyrophosphate phosphodiesterases (E-NPPs), ecto-nucleoside triphosphate diphosphohydrolases (ENTPDases), and adenylate kinases [1,2,3].

Pioneer studies described CD39 as adenosine triphosphate (ATP) diphosphohydrolase in vascular/endothelial cells, defining its crucial role in thromboregulation [4,5]. Together with the enzymatic activity of CD73, CD39 is responsible for a cascade in which ATP is converted into adenosine diphosphate (ADP) and cyclic adenosine monophosphate (cAMP), ultimately generating extracellular adenosine. The broad immunosuppressive effects of extracellular adenosine have been widely reported [6], and the immunosuppressive activities adenosine-mediated have been thoroughly reviewed [7,8].

The adenosine pathway occurs via type 1 purinergic receptors (A_1_, A_2A_, A_2B_, A_3_), which are G protein-coupled receptors (GPRCs). A_1_ and A_3_ receptors inhibit adenylate cyclase and cAMP generation. They are generally described as immune-promoting adenosine receptors [9]. In contrast, A_2AR_ and A_2BR_ are typically associated with a high level of immunosuppression, triggering intracellular cAMP accumulation [6,7,10]. In line with this, genetic deletion of the A_2A_ receptor was shown to induce tumor rejection in mice, providing a critical link between the adenosine pathway and tumor immunity [10]. Although the A_2B_ receptor has been described in tumor cells [11], both the A_2A_ and A_2B_ receptors are largely expressed in different immune cells, including myeloid and lymphoid compartments [12,13,14]. Under normal physiological conditions, concentrations of extracellular ATP (eATP) are negligible in tissue, i.e., 10–100 nM. However, under inflammatory conditions, and in response to diverse stimuli such as hypoxia/tissue injury or in tumors, high eATP levels (1–50 µM) may be detected [9,11,15].

Various cell types may release ATP into the microenvironment through deputed channels (i.e., pannexin or hemichannel connexins), or as result of necrosis from dying or stressed cells. Robust inflammatory signals are provided by high eATP levels through the engagement of P2 receptors (P2Y and P2X families), which, in turn, are critical for the activation of both innate and adaptive immune responses. The P2×7 receptor has been described as the most important in the context of inflammation [16]. It has been reported to be highly expressed in granulocytes, macrophages, dendritic cells, B and T cells, and in particular, in CD4^+^ T cells expressing FOXP3, the master regulator of the development and function of regulatory T cells (Tregs) [17]. The production of IL-18 and IL-1β as active forms is mediated by eATP via the P2X7 receptor, which triggers NLRP3 inflammasome activation [18].

CD39 upregulation is therefore an efficient mechanism developed by tumors to escape antitumor strategies by depleting the immune-stimulatory eATP in the tumor microenvironment (TME).

CD39 has been observed to be highly expressed in different human tumor types, such as renal cell carcinoma, ovarian cancer, sarcoma cancer, breast cancer, lymphoma, bladder cancer, colon cancer and melanoma [19,20,21,22]. Tumor cells can overexpress CD39 compared with normal cells; however, elevated levels have been also reported in endothelial cells, cancer-associated fibroblasts (CAF) and several immune subpopulations, particularly, natural killer (NK) cells, tumor-associated-macrophages (TAM) and tumor-infiltrating lymphocytes (TILs) including Tregs and CD8^+^ T cells [23,24,25,26,27,28]. High CD39 expression is considered a marker of poor outcome and disease progression [29,30,31].

eATP and extracellular adenosine levels are also regulated by CD73 expression, the ecto-enzyme converting AMP into adenosine. CD73 is frequently expressed in human tumors, particularly in tumor cells, CAF and endothelial cells, but also in myeloid cells, NK cells and T cells [32,33,34,35]. Most data described a strong correlation between elevated CD73 levels and unfavorable clinical outcomes, as was observed for CD39 [36,37,38,39]. Through CD39 and CD73 blockade, likely limiting the conversion of ATP/AMP into adenosine, many mouse, human and in vitro studies showed inhibition of both tumor growth and metastasis formation, generally associated with an increase of NK and/or CD8 T cell immune-mediated antitumor responses [21,25,33,40,41,42,43,44].

## 2. CD39 Expression and Functions in Conventional CD4^+^ T Cells and Tregs

CD39 has been primarily described as a (FOXP3^+^) Treg marker [24], whose hydrolysis of extracellular ATP is crucial in terms of their immunosuppressive functions. In vitro and in vivo studies on *ENTPD1*-deficient (CD39^-/-^) mice demonstrated an impairment in Treg suppressive functions. Accordingly, adenosine formation in murine and human Tregs is mediated by the coexpression of CD39 and CD73 [24,45]. Adenosine-triggered signals may increase intracellular cAMP levels, resulting in the transactivation of *ENTPD1* promoter, that, in turn, increases and stabilizes CD39 expression in Tregs [46]. The eATP signaling pathway, mediated by P2X7 receptors and phospho-Erk, may interfere with the differentiation and suppressive functions of Tregs. Indeed, eATP depletion in the TME limits the activation of HIF-1α factor, which is mainly responsible for the ubiquitination and degradation of FOXP3 [45]. In line with this, reduced numbers of CD39^+^FOXP3^+^ Tregs were observed in patients affected by multiple sclerosis (MS), indicating that CD39 expression in Tregs is critical in controlling inflammatory autoimmune disorders [24]. In contrast, circulating levels of CD39^+^CD25^+^ Tregs were elevated in cancer patients [47,48], while their low levels were associated with improved relapse-free survival in melanoma patients [49]. Accordingly, CD39^+^ Treg frequency and adenosine-mediated immunosuppression were significantly increased in head and neck patients [50]. Importantly, elevated levels of CD39, often concomitant with elevated levels of other suppressive/activation markers (i.e., OX40, PD1, CTLA-4) [51,52], were observed in tumor-infiltrating Tregs, which generally occurred in elevated number and frequency in solid tumors [51,53]. All these observations suggest the key role of CD39 in attenuating immune responses in cancer.

CD39 may also be expressed in conventional (c) FOXP3^–^ CD4^+^ T cells. For example, in squamous cell carcinoma patients, CD39^+^ cCD4^+^ T cells revealed tumor-specific antigen reactivity [54]. Interestingly, CD39-expressing cCD4^+^ T cells have often been associated with T helper (Th) 17 effector functions. Th17 cells are known to promote chronic inflammation and regulate commensal bacteria in the gut. Consistent with this, eATP production by commensal bacteria may support Th17 differentiation. Comparably, P2X7 receptor may favor Th17 conversion from Tregs [55]. On the other hand, CD39-expressing Th17 cells are associated with increased IL-10 production, and CD39^+^ Th17 cells predict poor clinical outcome in cancer patients [56]. Accordingly, CD39^+^ tumor-infiltrating Tregs may produce IL-17, in contrast to CD39^-^ tumor-Tregs, in colon cancer patients [51], supporting the hypothesis that CD39^+^ IL-17-producing cells play a determining role in cancer.

## 3. CD39 Expression and Functions in CD8^+^ T Cells

CD39 expression in CD8^+^ T cells has been recently reported. Pioneer studies described CD39 as a marker of exhaustion. Gupta and colleagues demonstrated that CD39 expression identified terminally exhausted virus-specific CD8^+^ T cells in HCV and HIV chronic infections [57]. More recently, Canale and colleagues reported that tumor-infiltrating CD39^high^ CD8^+^ T cells exhibited low TNF−α, IFN-γ and IL-2 production. CD39 expression was accompanied by coinhibitory receptors (i.e., LAG3, TIGIT, PD1, TIM3, 2B4) and associated with tumor growth [28]. CD39^+^ CD8^+^ T cells have been found in invaded lymph nodes and metastases [28], and are generally associated with poor survival, as in clear cell renal carcinoma [58]. In 2018, an elegant paper described, for the first time, that CD39 selectively marked tumor (neoantigen)-specific CD8^+^TILs, whereas those CD39^–^ comprised bystander CD8^+^ T cells which are able to recognize a wide range of viral epitopes [23]. Interestingly, CD39^+^CD8^+^ TILs were enriched in genes associated with cell proliferation and exhaustion, and displayed reduced TCR diversity, as typically observed in chronically antigen-stimulated T cell expansion [23]. In line with this evidence, many papers have described CD39^+^ CD8^+^ TILs as tumor-antigen specific and reactive cells [59,60]. These polyfunctional and protective roles are mostly associated with CD103 co-expression in several cancer types [23,44,60,61]. CD103 is indeed a marker of tissue resident memory CD8^+^ T cells, and its expression is frequently correlated with good progression, positive outcome and better survival in several cancers [60,62,63].

However, several groups have suggested that CD39-expressing CD8^+^ T cells showed regulatory properties [64,65]. Although CD8^+^ Tregs have been widely studied in the past 30 years, their markers have not yet been fully elucidated [64]. Our group and others showed that CD39 is potentially involved in mediating the suppressive abilities of tumor-infiltrating CD8^+^ Tregs. Isolated CD39^+^ CD8^+^ T cells were indeed found to be capable of suppressing T cell proliferation in vitro [44]. Moreover, CD39 counteraction significantly inhibited the suppressive capacities of CD8^+^ Tregs, highlighting its key role in mediating suppressive functions [66], as also described for CD4^+^ Tregs [24].

Taken together, these apparently contrasting data suggest that CD39 is upregulated by antigen-driven, activated CD8^+^ T cells in an attempt to regulate excessive immunopathologies by its intrinsic capacity to provide stimulated T cells with a suppression function. This regulatory mechanism may be beneficial in conditions in which it is necessary to switch off immune responses, for example, following recovery from infection. In contrast, it would also be detrimental in tumor conditions, i.e., by providing suppression capacity to tumor-specific CD39^+^ CD8^+^ T cells that are chronically stimulated by tumor antigenic persistence.

## 4. CD39 Regulation by Cytokines

While the vast majority of the data describe CD39 expression and its functions associated with an ex vivo phenotype in TME, less is known about the mechanisms by which it is upregulated in FOXP3^+^ Tregs, cCD4^+^ and CD8^+^ T cells under specific circumstances.

Circulating cCD4^+^ T cells show negligible levels of CD39. In contrast, blood Tregs from healthy donors (HD) may express around 0–5% of CD39^+^ cells; this frequency may increase in cancer patients, reaching about 5–20% [53,67].

CD39 frequency may be highly increased in TI-Tregs and TI-cCD4^+^ cells compared to its circulating counterparts, suggesting that peculiar factors in TME can contribute to CD39 levels.

In vitro studies have shown that the combination of TCR engagement and IL-2 may increase CD39 expression. Additionally, TGF-β was found to be particularly likely to induce the expansion of CD39^+^ Tregs, supporting the hypothesis that tumor-derived factors play important roles in CD39 upregulation in Tregs [67]. Comparably, the TGF-β/SOX4 signaling pathway, with the participation of ROS-driven autophagy, was shown to mediate CD39 expression in Tregs [68]. IL-27 signaling was found to directly drive CD39 expression in Tregs by a STAT-1-dependent mechanism in mice studies. Indeed, mice lacking the IL-27 EBI3 subunit or IL-27Rα failed to display CD39 upregulation on tumor-infiltrating Tregs [69]. IL-35 has also been described as being responsible for CD39 induction in Tregs [70].

Consistent with CD39 levels in peripheral cCD4^+^ T cells, negligible CD39 levels are observed in circulating CD8^+^ T cells in both healthy subjects and cancer patients [28,44]. However, the frequency of CD39^+^ CD8^+^ TILs is enhanced in many cancer types [23,28,44,60].

As for FOXP3^+^ Tregs and cCD4^+^ T cells, upon TCR stimulus in vitro, blood CD8^+^ T cells overexpress CD39 in both healthy subjects and cancer patients [28,71], as well as in murine CD8^+^ T cells [72]. Although TCR engagement was sufficient to upregulate CD39, IL-6 and IL-27 exposure further promoted CD39 expression in both human and murine CD8^+^ T cells [28]. IL-6 has been also shown to induce CD39 expression in tumor-infiltrating NK cells in esophageal squamous cell carcinoma, suggesting a broad IL-6-mediated mechanism regulating CD39 expression in several immune cells [73]. Among the factors involved in CD39 upregulation in CD8^+^ T cells, IL-12 and IL-4 have been described as important players [65].

## 5. CD39 Regulation by Genetic Factors

In humans, the number of circulating T cells is, in part, heritable, underlying the importance of genetic elements in balancing immune responses [74]. Recently, a complex network of genetic variants governing the regulation of immune cell levels in health and disease has been defined [75]. Additionally, the genetic structure of Tregs, which is largely determined by several single nucleotide polymorphisms (SNPs), has also been described [76].

SNP at the position rs10748643, is responsible for different CD39 expression levels in T cells. For the first time, this observation was made in Tregs, in which the A allele determined low CD39 levels, compared to G subjects, that instead showed higher CD39 levels. Subjects with the GG variant correlated with better control of inflammatory responses and with less IL-17 and IFN-γ production by pathologic effector cells, as compared with AA subjects [77]. In line with this finding, SNP associated with low CD39 expression was linked to increased susceptibility to Crohn’s disease [78]. Other studies have described the impact of rs10748643 SNP on Tregs in regulating autoimmune disorders, infections and cancers [51,77,79,80,81], as well as in graft-versus host disease in a humanized mouse model [81]. A different CD39 gene polymorphism, associated with low CD39 expression, was additionally correlated with slower HIV progression [82]. Remarkably, the different genetic variants determined the frequency of tumor-CD39^high^ Tregs, potentially balancing the immunosuppressive-mediated Treg responses in colon rectal cancers [51].

SNP at position rs_10748643 was also correlated with a lower or absent CD39 cell surface expression in cCD4^+^ T cells. AA subjects were less predisposed to upregulate CD39 by anti-CD3/CD28 stimulation, as compared with AG or GG individuals, thus influencing an in vivo T cell response to vaccination. Indeed, individuals with the AA variant showed higher specific-T cell response to different strains of the influenza virus [83].

Interestingly, CD39 resulted a negative checkpoint inhibiting the generation of T follicular helper (Tfh) cells. The study demonstrated that BCL6 (lineage-determining transcription Tfh factor) was able to inhibit CD39 expression. Additionally, increased Tfh frequencies were observed in subjects with a SNP preventing/limiting *ENTPD1* transcription. Accordingly, by reducing CD39 expression, Tfh generation and germinal responses were enhanced [84].

Even if rs_10748643 SNP has been widely studied in Tregs and cCD4^+^ T cells, we recently observed the same impact on CD8^+^ TILs. We demonstrated that circulating CD8^+^ T cells from AA individuals were less susceptible to CD39 upregulation upon TCR engagement in vitro. Moreover, SNP influenced CD39 levels in TI-CD8^+^ cells, balancing CD8^+^ T cell responses in colon rectal cancer [44].

## 6. Targeting Adenosine Pathway, CD73 and CD39 as Anti-Tumor Strategy

An increase in the number of clinical trials targeting adenosine pathway components has been observed in recent years. Data about the efficacy of this approach are still limited, since most trials are ongoing or are in the early phases of development.

Each member of the adenosine signaling pathway could be potentially considered a distinct therapeutic opportunity; however, each could be susceptible to compensatory effects, leading to the accumulation of adenosine. Importantly, the unique feature of CD39 inhibition is a dual impact, i.e., in maintaining low pro-inflammatory eATP levels and reducing adenosine accumulation; indeed, pharmacologic inhibition of CD73-mediated AMP processing or inhibition via A_2A_ address only the reduction of adenosine accumulation. Of note, CD39 inhibition demonstrated antitumor efficacy as single-agent, whereas the use of two different anti-CD73 antibodies or an A_2A_ small-molecule inhibitor only delayed tumor growth in MC38 mouse models. Additive affects have therefore been observed when targeting both CD39 and CD73 in combination [85], or when combining anti-CD73 with A_2A_ small-molecule inhibitors [33].

**Figure 1:**
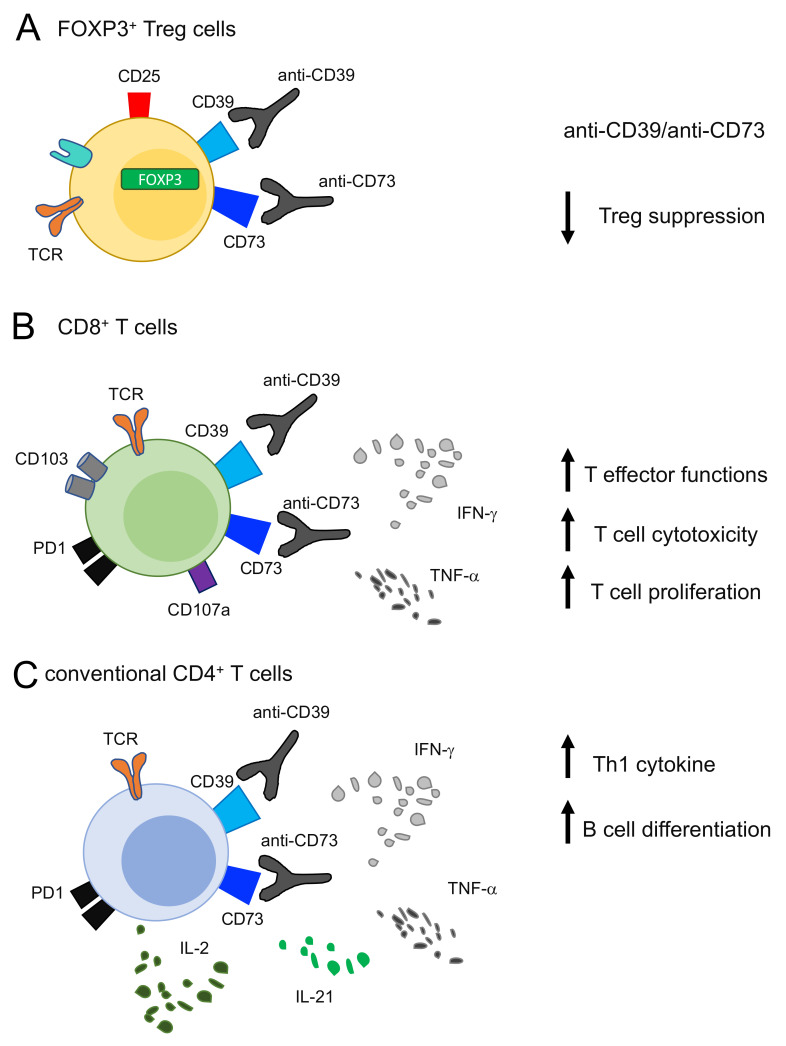
Effects of targeting CD39 on T cells. (**A**) CD39 or CD73 blocking by pharmacological compounds (i.e., POM1 and ARL7156 or small-molecule inhibitors) or by using antibodies in in vivo systems decreases the suppressive activities of FOXP3^+^ Treg cells. (**B**) Targeting CD39 or CD73 in CD8 T cells effects an increase in effector and cytotoxic T cell functions, mediated by IFN-γ and TNF-α production and CD107a upregulation. (**C**) Targeting CD39 or CD73 in CD4 T cells increases Th1 cytokine (IFN-γ, TNF-α and IL-2) and IL-21 production, which (was recently found to be) is responsible for B cell differentiation.

The inhibition of CD73 and CD39 has similar effects on T cells. Different studies have shown that anti-CD73 antibodies could restore antitumor CD8^+^ T cell responses, as well as reducing the suppressive activities of Treg; see Figure 1 [42,86,87].

Anti-CD73 small molecule inhibitors were shown to reduce ovarian cancer progression and increase survival in mice [86]. Similarly, Häusler and colleagues demonstrated that in human primary ovarian cancer cells and cell lines, CD73 and/or CD39 siRNA or small-molecule inhibitors enhanced NK-mediated cytotoxic T-cell activity and CD4^+^ T cell proliferation [22]; see Figure 1. Because CD73 is expressed in many host cell types such as endothelial cells or CAFs, CD73 inhibition could have nonhematopoietic effects, i.e., a broad effect which is less observed when CD39 is targeted. Accordingly, many groups have collectively demonstrated that CD73-deficient mice are resistant to growth and metastasis formation in immunogenic tumors, and that CD73 deficiency in both hematopoietic and nonhematopoietic cells is required to limit tumor growth [43,88,89]. It is noteworthy that host CD73 deficiency or blocking were shown to increase tumor antigen specific-T cell homing to tumors. It has been demonstrated that high levels of CD73 in endothelial cells limit the migration of tumor-specific CD8^+^ T cells at the tumor site [89].

Most findings about the role of CD39 in antitumor immunity and tumor growth were the result of studies on CD39-deficient mice. Genetic depletion of *Entpd1* in mice reduced metastatic lesions by active NK-mediated surveillance [27,90].

More recently, other researchers have shown the effects of monoclonal antibodies targeting CD39 in several tumor models [26,85]. Anti-CD39 treatment suppressed metastasis by enhancing IFN-γ^+^ and/or CD107a^+^ TI-NK cells in a lung cancer mouse model [26]. In line with this, antibodies targeting human CD39/CD73 membrane-associated or soluble forms were shown to reduce tumor growth by boosting T-cell antitumor responses in syngeneic mouse models implanted in different tumor cell lines (MC38, MCA205, B16F10) [85]. An additional study confirmed that triggering the eATP-P2X7 pathway by anti-CD39 improved tumor T cell infiltration and rescued anti-PD1 resistance by exhausted T cells [25]. These studies were then corroborated by the use of ectonucleotidase inhibitors, POM1 and ARL7156, i.e., pharmacological compounds which are frequently used in in vivo and in vitro studies [44,91,92]. Although data derived from these pharmacological compounds should be interpreted with caution due to their lack of ectonucleotidase inhibitor specificity, many findings showed that ARL67156 and/or POM-1 increased IL-21 and IL-2 production in cCD4^+^ T cells by restoring their ability to induce B cell differentiation [83] (Figure 1). Additionally Tfh generation and germinal center responses were enhanced by reducing CD39 expression via cAMP/PKA/p-CREB pathway inhibition [84]. Inhibition of CD39 activity via the ARL CD39 inhibitor contributed to protecting cCD4^+^ T cells from apoptosis and to increasing effector cCD4^+^ T cell differentiation and survival [83]. Recently, we described that POM1-treated CD8^+^ TILs restored IFN-γ and CD107a and decreased PD1 expression, compared to CD39^-^ CD8^+^. CD39 inhibition mediated by POM1 may therefore shift the CD8^+^ T cell balance toward a functional effector phenotype in cancer [44] (Figure 1).

Given the abundant expression of CD39 on Tregs, they are certainly one of the major targets of CD39 inhibition. Firstly, *CD39*-null mice showed an impairment in Treg suppressive activities in melanoma and colon cancer models in both in vitro and in vivo experiments [93,94]. POM1-mediated CD39 inhibition was able to abrogate Treg suppressive capacitates by diminishing NK and T cell inhibition [94] (Figure 1). Similarly, knock down of CD39 by using a CD39 antisense oligonucleotide in vivo reduced the number of tumor-infiltrating CD39^+^ Tregs; as a consequence, the ratio of CD8^+^ T cells to Tregs in tumors was significantly improved [40].

## 7. CD39 Targeting for Immunotherapy Strategies

Targeting CD39 by knockdown of the *Entpd1* gene led to the dysregulation of many physiological processes in mice, including glucose tolerance, angiogenesis, thrombo-regulation, coagulation and inflammatory responses. CD39^-/-^ null mice showed impaired hepatic insulin sensitivity, accompanied by high levels of insulin, IL-6, IL-1β, TNF-α and IFN-γ in the serum. These data suggest a robust link between CD39 and inflammation in regulating metabolic responses. Consistently, a mouse model with total CD39 deletion showed high susceptibility to murine colitis, confirming the key role of CD39 in regulating susceptibility to inflammatory bowel diseases [78,95,96]. In line with this, an interesting observation was made in human siblings with a homozygous mutation in the *ENTPD1* gene. They were characterized by a stop-codon mutation, which presented low ATP activity and low AMP production; they went on to develop neurological and gastrointestinal disorders [97]. Given the impact of CD39 deletion in vivo resulting in the dysregulation of many physiological processes, nowadays, selective antibodies are designed to specifically target CD39 in order to inhibit its activities [27,85].

While the vast majority of clinical trials involving adenosine pathways have mainly evaluated the use of anti-CD73 mAb and A2aR/A2bR antagonists [9,98], three agents targeting CD39 were recently developed and included in clinical trials in patients with advanced solid tumors. TTX-030 is one of the newest human monoclonal antibodies, developed by Tizona Therapeutics. Two randomized clinical trials, NCT03884556 and NCT04306900, respectively posted in March 2019 and March 2020, sought to study the safety, tolerability, pharmacokinetics and anti-tumor activity of the TTX-030 antibody as a single therapy and/or in combination with an approved anti-PD-1 immunotherapy (Pembrolizumab/Budigalimab) and/or standard chemotherapies (Docetaxel, Paclitaxel and Gemcitabine). Similarly, a CD39 blocking antibody (IPH520) was recently developed by Innate Pharma and entered in a recent clinical trial (NCT04261075) with the objective of studying IPH5201 as a monotherapy or in combination with anti-PDL1 (Durvalumab) and/or anti-CD73 (Oleclumab).

## 8. Future Perspectives

Adenosine signaling pathway components are currently considered as important targets in the treatment of various cancers [9,98]. Although monotherapies may achieve good results, almost all ongoing clinical trials involving blocking the adenosine pathways include arms in combination with standard chemotherapies or immune check point blocked (ICB) therapies.

Despite the remarkable effects of ICB therapies in several cancers [99,100,101], only around 30% of patients responded to ICB as a monotherapy [99,100,101,102], supporting the notion that a considerable portion of patients fail to mount protective/beneficial responses. One of the mechanisms associated with immunosuppression following ICB therapy could be dying tumor cells, which would provide immunosuppression due to eATP/adenosine release. Therefore, combining ICB with classical chemotherapies and/or adenosine/CD73 and CD39 blockade may improve the efficacy of these treatments, as well combining ICB with one or more blockade targeting adenosine components. For example, the combination of anti-CD73 with either anti-PD1 or anti-CTLA-4 may have beneficial effects [98,103,104]. Given the advances in CAR-T technologies as new tools to fight cancer, T cells could be alternatively engineered to be deficient in CD73/CD39 or adenosine receptors [105,106], improving their antitumor effector functions.

Altogether, these observations highlight the potential importance of targeting CD73, adenosine components and/or the CD39 pathway. In the near future, we will be able to comprehensively compile data from ongoing clinical trials testing anti-CD73, anti-CD39 and/or other adenosine components alone or in combination with ICB.

## Abbreviations

regulatory T cells (Tregs)
conventional CD4 T cells (cCD4^+^ T cells)
cancer-associated fibroblasts (CAF)
multiple sclerosis (MS)
tumor-infiltrating (TI)
tumor-infiltrating lymphocytes (TILs)
natural killer (NK)
tumor-associated-macrophages (TAM)
tumor-microenvironment (TME)
adenosine triphosphate (ATP)
adenosine diphosphate (ADP)
cyclic adenosine monophosphate (cAMP)
extracellular ATP (eATP)
immune check point blocked (ICB)
single nucleotide polymorphism (SNP)
